# Pain self-management plus activity tracking and nurse-led support in adults with chronic low back pain: feasibility and acceptability of the problem-solving pain to enhance living well (PROPEL) intervention

**DOI:** 10.1186/s12912-023-01365-y

**Published:** 2023-06-25

**Authors:** Wanli Xu, Yiming Zhang, Zequan Wang, Susan G Dorsey, Angela Starkweather, Kyounghae Kim

**Affiliations:** 1 University of Connecticut School of Nursing, Storrs, Connecticut USA; 2 Center for Advancement in Managing Pain, University of Connecticut, Storrs, Connecticut USA; 3 Department of Statistics, University of Connecticut, Storrs, Connecticut USA; 4 School of Nursing, University of Maryland, Baltimore, USA; 5 University of Florida College of Nursing, Gainesville, USA; 6 College of Nursing, Korea University, 145 Anam-ro, Seongbuk-Gu, Seoul, South Korea; 7 Nursing Research Institute, Korea University, Seoul, South Korea; 8 BK21 FOUR R&E Center for Learning Health Systems, Korea University, Seoul, South Korea

**Keywords:** Activity tracking, Chronic low back pain, Self-management

## Abstract

**Background:**

Chronic low back pain can lead to individual suffering, high medical expenditures, and impaired social well-being. Although the role of physical activity in pain management is well established, the underlying mechanisms of biological and clinical outcomes are unknown. This study aimed to assess the feasibility and acceptability of a pain self-management intervention, Problem-Solving Pain to Enhance Living Well, which employs wearable activity tracking technology and nurse consultations for people with chronic low back pain.

**Methods:**

This one-arm longitudinal study recruited 40 adults aged 18–60 years with chronic low back pain. Over 12 weeks, participants watched 10 short video modules, wore activity trackers, and participated in nurse consultations every 2 weeks. At baseline and the 12-week follow-up, they completed study questionnaires, quantitative sensory testing, and blood sample collection.

**Results:**

Forty participants were recruited, and their mean age was 29.8. Thirty-two participants completed the survey questionnaire, quantitative sensory testing, Fitbit activity tracker, and bi-weekly nurse consultation, and 25 completed the evaluation of biological markers. The overall satisfaction with the Problem-Solving Pain to Enhance Living Well video modules, nurse consultations, and Fitbit in pain management was rated as excellent. No adverse events were reported. Between the baseline and 12-week follow-up, there was a significant decrease in pain intensity and interference and an increase in the warm detection threshold at the pain site.

**Conclusions:**

Despite concerns about the participant burden due to multidimensional assessment and intensive education, the feasibility of the Problem-Solving Pain to Enhance Living Well intervention was favorable. Technology-based self-management interventions can offer personalized strategies by integrating pain phenotypes, genetic markers, and physical activity types affecting pain conditions.

**Trial registration:**

This pilot study was registered with ClinicalTrials.gov [NCT03637998, August 20, 2018]. The first participant was enrolled on September 21, 2018.

## Background

Chronic low back pain (cLBP) is one of the most prevalent pain conditions in the U.S., affecting 20.4% of adults [1]. Globally, cLBP is the most common cause of years lived with disability, affecting 64.9 million people worldwide [2, 3]. The direct medical costs of cLBP and indirect expenses related to disability impose a substantial economic burden on individuals and the society. In the U.S., the estimated annual expenditure related to spinal pain (combining neck and/or low back pain) is $134.5 billion [4]. While most episodes of acute low back pain resolve in 4–6 weeks, approximately 32% of individuals transition to cLBP and require ongoing care, constituting a majority of the annual expenditures related to spinal pain [5]. Previous studies have reported peripheral and central nervous system sensitization are involved in the functional alterations in cLBP, which can be captured by quantitative sensory testing (QST) [6]. Identifying effective interventions to facilitate cLBP management and preserve physical and social functioning is critically important for population health, quality of life, and efforts to reduce costs from the overuse of unwarranted diagnostic and treatment approaches [7].

Current practice guidelines for cLBP [8, 9] as well as the National Pain Strategy [10], emphasize that pain self-management is the first-line standard of care but provide little guidance on the elements that should be addressed in a self-management intervention. Research on self-management continues to advance with more extensive self-management-specific frameworks [11, 12]. None of the studies designed and implemented interventions that delineated theory-driven self-management elements for cLBP management [13]. To address this gap, our team used a person-centered approach to develop a theoretically based self-management intervention called Problem-Solving Pain to Enhance Living Well (PROPEL) [14]. The PROPEL incorporates evidence-based strategies that are effective in improving pain and somatosensory function [15]. Specifically, PROPEL was guided by the Individual and Family Self-Management Theory (IFSMT) which delineates modifiable context and process factors [12] and has been verified in the context of chronic conditions, such as diabetes [16] and heart failure [17]. According to IFSMT, self-management knowledge and beliefs, self-regulation, and social facilitation are affected by condition-specific factors along with individual, family, and environmental factors, which in turn influence proximal (self-management behaviors) and distal outcomes (perceived well-being). PROPEL also incorporates evidence-based, standard-of-care methods to promote physical activity among individuals with pain, and tools to improve knowledge, skills, and confidence in coping with cLBP. The intervention and study protocol details have been previously reported [14], and the study was registered in a clinical trial database [NCT03637998].

## Methods

### Aim

The aim of this study was to examine the feasibility, acceptability, and preliminary efficacy of the PROPEL intervention using pre- and post-test data from a single-arm longitudinal study that enrolled 40 participants with cLBP.

### Design

This longitudinal study enrolled 40 participants with cLBP who received the PROPEL intervention, nurse-led self-management plus activity tracking. A control group was not included because the main focus of the evaluation was to assess differences in the intervention components from pre- to post-testing.

### Study settings and participants

Participants were recruited from the local communities surrounding a research-intensive university in the New England region using active and passive strategies that included (1) contacting pain registry participants maintained by the research team; (2) distributing flyers in the local community and outpatient health clinics; and (3) placing advertisements in local newspapers, websites, and social media (Facebook and Instagram), which instructed interested volunteers to contact a study-designated phone or email address.

The inclusion criteria were as follows: (1) English-speaking adults with cLBP aged 18 to 60 years; (2) having no other type of chronic pain conditions; and (3) access to a computer or smart mobile device with Internet connection. The exclusion criteria were as follows: (1) any history of comorbidities that influence sensorimotor function, including multiple sclerosis, cancer, spinal cord injury, or diabetes; (2) history of spinal surgery in the previous year; (3) presence of neurological deficits, including lower extremity weakness; (4) history of bowel or bladder dysfunction; (5) positive Romberg test or sciatica upon leg raise; (6) current pregnancy or within 3 months postpartum; and (7) hospitalization in the past 6 months due to mental health disorders.

### PROPEL intervention

Details of the PROPEL intervention have been reported elsewhere [10]. In brief, PROPEL consists of 10 online self-management modules, activity tracking, and biweekly nurse consultations during the 12-week intervention period. The modules offer factual information on low back pain neurophysiology, strategies for promoting self-regulation and problem-solving, and instructions on managing pain while maintaining regular functions.

### Procedures

Trained research assistants (RA) screened the interested volunteers during a confidential phone call to determine their eligibility. Eligible participants were scheduled for a baseline visit to discuss the study and answer any questions. Informed consent procedures were followed, and written consent was obtained from each participant by the study staff.

The enrolled participants were immediately scheduled for their baseline data collection visit, which involved a physical examination, completing study questionnaires and QST, as well as the collection of blood samples [14]. The QST is used to measure pain sensitivity and uses standardized stimuli to test the nociceptive systems in the periphery and central nervous systems [6, 18]. Seven tests measuring 13 functional sensory pathways are grouped as follows [18]: “(1) thermal detection thresholds for the perception of cold, warm, and paradoxical heat sensation; (2) thermal pain thresholds for cold and hot stimuli; (3) mechanical detection thresholds for touch and vibration; and (4) mechanical pain sensitivity including thresholds for pinprick and blunt pressure, stimulus/response-functions for pinprick sensitivity and dynamic mechanical allodynia, and pain summation to repetitive pinprick stimuli (wind-up like pain).” QST was performed in both the pain and control sites. The medial side of the non-dominant forearm was used as a control. Venous blood samples were collected in one 2.5 ml PAXGENE tube (QIAGEN, Hilden, Germany) and were immediately transported, processed, and stored at -80° laboratory freezer conditions for RNA sequencing. If the blood draw was unsuccessful, the participants were required to provide a buccal cell sample for genetic testing. The analysis of gene expression will be reported separately and was not included in this manuscript.

Following data collection, study staff assisted participants in setting up and syncing a Fitbit device on their personal cell phone, and were then given instructions on how to maintain and wear it until their 12-week follow-up appointment. Participants were informed that they would receive an email link to a PROPEL module daily for the next 10 days, and were instructed to watch the modules and be prepared to discuss the content during the nurse consultation visit. Nurse consultations were delivered to participants via phone interviews at weeks 2, 4, 6, 8, and 10. At week 12, the nurse consultations were delivered in person. After the baseline visit, participants were scheduled for their 12-week follow-up visit, in which they completed the study questionnaires, QST, and a blood draw.

Participants in the study received a $20 gift card for the baseline visit and $40 for the final visit. Upon completion of both questionnaires and nurse consultations at weeks 2, 4, 6, 8, and 10, participants were given a $10 gift card. In addition, they received $5 biweekly for charging and syncing their Fitbit. If participants completed all follow-up questionnaires and intervention components, they were provided with a total of $140. The Fitbit was given to participants to keep.

### Measures of feasibility, acceptability, and preliminary efficacy

#### Feasibility and acceptability benchmarks

*Feasibility of recruitment:* The enrollment rate was determined by the number of participants who consented divided by the total number of individuals who made initial contact with the study team (signed up on social media advertisements or made an initial phone call) and met the inclusion criteria.

*Acceptability* was assessed using the retention and attrition rates. This feasibility benchmark was defined as acceptable if 75% of the participants completed the baseline and the 12-week follow-up visits.

*Adherence to Fitbit:* We evaluated each participant’s compliance rate with wearing the Fitbit device by the proportion of time with non-zero intensity during the awake time. A zero intensity in 1 h indicated that the participant did not wear the Fitbit device during that hour. Thus, a lower proportion of non-zero intensity time showed a higher compliance rate when wearing the Fitbit.

*Adherence to surveys:* Adherence to longitudinal self-reported data collection was evaluated by the mean percentage of completed biweekly REDCap survey questionnaires and consultations. Questionnaires were excluded from the analysis when entirely missing.

*Adherence to nurse consultation:* Participants had opportunities to express their concerns regarding their symptoms and self-management skills via bi-weekly consultations with nurse research staff. The nurse and the participant exchanged ideas on possible solutions to these challenges. We also measured the frequency of participants practicing pain self-management skills, including deep breathing, muscle relaxation, and guided imagery, throughout the study period. We considered it excellent if the proportion of participants completing consultation sessions was ≥ 80% and good if it was ≥ 75%.

*Adherence to biospecimen and QST measures:* The feasibility of biospecimen collection was measured by the percentage of blood samples collected in the total collection attempts. The feasibility of the QST procedure was assessed based on the percentage of participants who had completed the QST protocol. Data collection was performed at baseline and at 12-week follow-up. We obtained a buccal cell sample if a blood sample could not be collected because of small or hard-to-find veins so that genetic assays could be included.

*Program satisfaction:* PROPEL was assessed using a participant satisfaction questionnaire that captured the extent to which the intervention met the participants’ needs and preferences. This 10-point Likert scale ranges from 0 to 10, with 0 being the lowest level of satisfaction and 10 being the highest. We considered program satisfaction to be excellent if the proportion of participants rating PROPEL was ≥ 80%, and good if it was ≥ 75%.

*Program safety:* The safety of the intervention was determined by recording self-reported adverse events during consultation phone calls and in-person visits. Program safety was considered excellent if no adverse events directly linked to PROPEL participation were reported and good if minimal to mild adverse events related to PROPEL occurred in < 5% of the study participants.

#### Pain (brief pain inventory [BPI]-SF, PROMIS-pain intensity, QST)

The BPI-SF is a reliable and valid measurement to assess participants’ average pain intensity and average interference with functioning due to pain, including activity, emotion, relationships with others, employment, and sleep [9]. A composite mean score of the BPI pain intensity items, including “worst,” “least,” “average,” and “now,” was generated, indicating BPI pain severity. BPI interference was estimated by calculating the mean interference score with seven daily activity domains. Additionally, the PROMIS-Pain Intensity measure is recommended as a supplemental instrument for NIH-funded research.

The QST is a non-invasive technique used to assess somatosensory functions and pain perception through the application of standardized thermal and mechanical stimuli [14]. Thirteen functional sensory pathways were evaluated to detect abnormalities in large A-beta and small C- and A-delta sensory fibers in the peripheral and central nervous systems [14]. Detailed information regarding the administration of the QST protocol among individuals with cLBP has been published elsewhere [6].

#### Physical activity (fitbit, godin-leisure questionnaire)

The Fitbit Flex 2 auto-detected the participants’ activity and recorded the minute-level Metabolic Equivalents (MET) data and physical activity category data, such as sedentary time, lightly active time, fairly active time, and very active time. For each participant, we calculated the average MET level per minute during the effective wearing time in each week and use it as a weekly level continuous outcome in Fitbit data analysis. The effective wearing time was approximated by excluding the unoccupied time, which was identified by screening each participant’s data based on a 30-min moving window. If the minimum MET level was constantly recorded over the 30-min window, the Fitbit device was considered unoccupied.

However, true sedentary time, sleeping time, and unoccupied time were all recorded as sedentary time due to the limitation of the Fitbit Flex 2. If a participant did not wear the Fitbit device continuously, the duration of different levels of active time recorded by Fitbit Flex 2 did not reflect the true activity variability. Since this type of “missing” data cannot be identified in the Fitbit data set, the statistical missing data algorithm would not be applied. Therefore, more accurate and reliable measurements of physical activity are required for studies based on data collected using the Fitbit Flex 2.

We processed Fitbit data using minute-level MET data to approximate the effective wearing time for each participant. If the device did not move or was not worn by the participant, it recorded a minimum MET level of 1.0. We identified the unoccupied time of each participant’s Fitbit device by screening the data based on a 30-min moving window. If the minimum MET level was constantly recorded over the 30-min window, the Fitbit device was considered unoccupied. By excluding the estimated unoccupied time, we were able to estimate the duration of the effective time when the participants were wearing the Fitbit device. The weekly average MET level during the effective wearing time was used as the physical activity measure in Fitbit data analysis.

The Godin Leisure-Time Exercise Questionnaire is a reliable and valid measure to assess the number of strenuous, moderate, and mild intensity leisure-time physical activities for at least 15 min a week [19] in patients with cLBP. The weekly leisure activity score was calculated by multiplying nine, five, and three for strenuous, moderate, and mild activities, respectively.

### Statistical analysis

Statistical analyses were conducted using the SPSS 27 and R 4.0.3. Feasibility and acceptability were assessed using descriptive statistics. Summary statistics for baseline characteristics, self-reported pain, self-management skills, and physical activity were reported at each time point. To examine the preliminary efficacy of the PROPEL intervention, we performed a paired two-sample t-test on pain and self-management skills outcomes at baseline and visit seven (12-week follow-up visit). Shapiro-Wilk test was conducted to check the normality of the pre-post difference of each variable. If the normality assumption did not hold, paired Wilcoxon sign-rank test was used for testing. We calculated the effect size for the pre- and post-pain changes and QST measurements using Cohen’s D. We summarized the longitudinal Fitbit data from baseline to the 12th week and Godin-Leisure measures using descriptive statistics and trajectory plots.

### Ethical considerations

Prior to participant recruitment, this study was approved by the Institutional Review Board (IRB# H18-086).

## Results

### Demographic and clinical characteristics

The summary statistics of the demographic and clinical characteristics are presented in Table 1. The participants were predominantly female (62.5%), white (67.5%), non-Hispanic or Latino (87.5%), never married (67.5%), and had college or undergraduate education (67.5%). The average age was 29.8 (SD = 11.7) years, and the average BMI was 26.8 (SD = 7.0). Individuals with less than 150 min of moderate physical activity were eligible for this study, and 62.5% of the participants reported engaging in some form of physical activity 1–3 days per week. Approximately 45% of the participants had low back pain for 1–5 years, and 55% reported pain frequency on at least half of the days over the past six months. Nearly 10% of the participants used opioid analgesics, and 32.5% used exercise therapy for cLBP.

**Table 1:** Descriptive table for demographic and clinical characteristics (N = 40)

Demographic and Clinical Characteristics		Descriptive Statistics	
		Mean	SD
Age		29.8	11.7
BMI		26.8	7.0
		Frequency	Proportion (%)
Gender	Male	15	37.5
	Female	25	62.5
Race	White	27	67.5
	Black or African American	4	10
	Asian	6	15
	Not reported	3	7.5
Ethnicity	Not Hispanic or Latino	35	87.5
	Hispanic or Latino	4	10
	Not reported	1	2.5
Education Level	High school or below	3	7.5
	College and undergraduate	27	67.5
	Graduate school	10	25
Employment Status	Working now	19	47.5
	Unemployment	3	7.5
	Student	18	45
Marital Status	Married	8	20
	Never married	27	67.5
	Others	5	12.5
Alcohol Use	Never	11	27.5
	Occasional	22	55
	Weekly or daily	7	17.5
Have you drunk or used drugs more than you meant to?	Never	30	75
	Rarely	9	22.5
	Sometimes	1	2.5
Exercise amount	None	10	25
	1–3 days/week	25	62.5
	4–5 days/week	5	12.5
How long has LBP been an ongoing problem for you?	3–6 months	3	7.5
	6 months-1 year	9	22.5
	1–5 years	18	45
	More than 5 years	10	25
How often has LBP been an ongoing problem for you over the past 6 months?	Every day	13	32.5
	At least half of the days	22	55
	Less than half of the days	5	12.5
Using Opioid painkillers	Yes	4	10.0
	No	36	90.0
Using exercise therapy	Yes	13	32.5
	No	27	67.5

Abbreviations: BMI, body mass index; LBP, low back pain

### Feasibility and acceptability

**Fig. 1:**
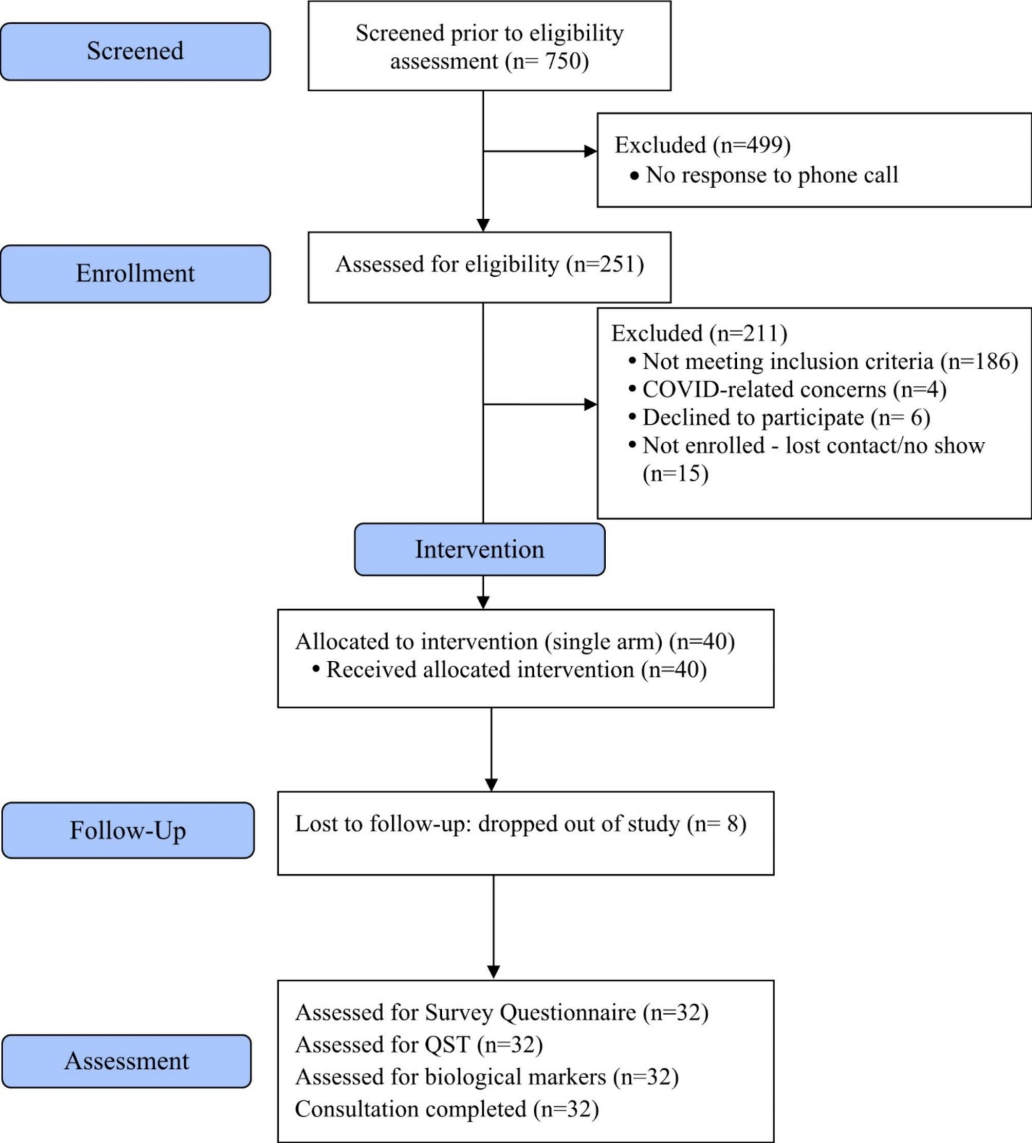
Study process Abbreviations: QST, quantitative sensory testing

Figure 1 displays the consort study diagram of this single-arm trial. Of the 750 individuals who had initial contact, 499 no longer responded to our team’s phone call; therefore, we could not complete the eligibility assessment. Of the 251 individuals assessed for eligibility, 186 did not meet the inclusion criteria. The most common reason for participants not meeting the inclusion criteria was having other types of chronic pain conditions, a history of spinal cord injury or spinal surgery, or neurological deficits. Among the 65 eligible participants, four could not attend a baseline visit because of possible COVID symptoms. Six participants declined, and 15 did not show up for baseline assessment. Of the 40 who initiated the data collection process, eight dropped out of the study. Thirty-two participants (80%) completed the assessment of the survey questionnaire, QST, biological markers, and nursing consultation. The overall enrollment rate was 61.5% (40/65), with 83% (25/30) in the pre-COVID period (09/2018–03/2020) and 42.8% (15/35) in the post-COVID period (10/2020–12/2021). Four participants (10%) withdrew from the study because of time conflicts and personal circumstances. Four participants (10%) were lost to follow-up despite multiple efforts. The overall retention rate was 80%, and the attrition rate was 22% for both pre- and post-COVID).

### Intervention/consultation (video-watching, consultation, and participant utilization of self-management strategies)

Thirty-seven participants (92.5%) watched the 10 video modules online, among whom six participants (16.2%) watched them a day after they received the modules, 21 participants (56.7%) watched them within a week, seven participants (18.9%) watched them within a month, and three participants (8.1%) completed them within more than a month. Participants found that the video modules were beneficial in providing information on their pain self-management (86.2%) and motivated them to engage in better pain self-management efforts (82.7%). Among the 10 videos, guided imagery and stretching were rated as the most favorable. Satisfaction with the overall quality of the videos (audio, pace, and organization) was rated as satisfied or very satisfied by 92.5% of the sample.

Thirty participants (75%) completed six nurse consultation sessions, with consultation durations ranging from 8 to 20 min. Of the total sample, 90% successfully provided goals to better manage their cLBP symptoms and learned problem-solving skills with nurses during the consultation, including aerobic and resistant physical activities, symptom management, medication management, diet/weight control, and stress management. With sufficient and understandable nurse consultation, participants could utilize self-management strategies to manage their pain over the course of this study. Satisfaction with nurse consultations was rated as satisfied to very satisfied by 89.6% of the sample, who reported that nurses were willing to listen to them, and participants were satisfied with respectful, sufficient, and understandable information. Participants reported that the nurse consultations provided a better understanding of decision-making (89.3% were satisfied to very satisfied) and pain self-management (92.9% were satisfied to very satisfied).

### Fitbit compliance

**Fig. 2:**
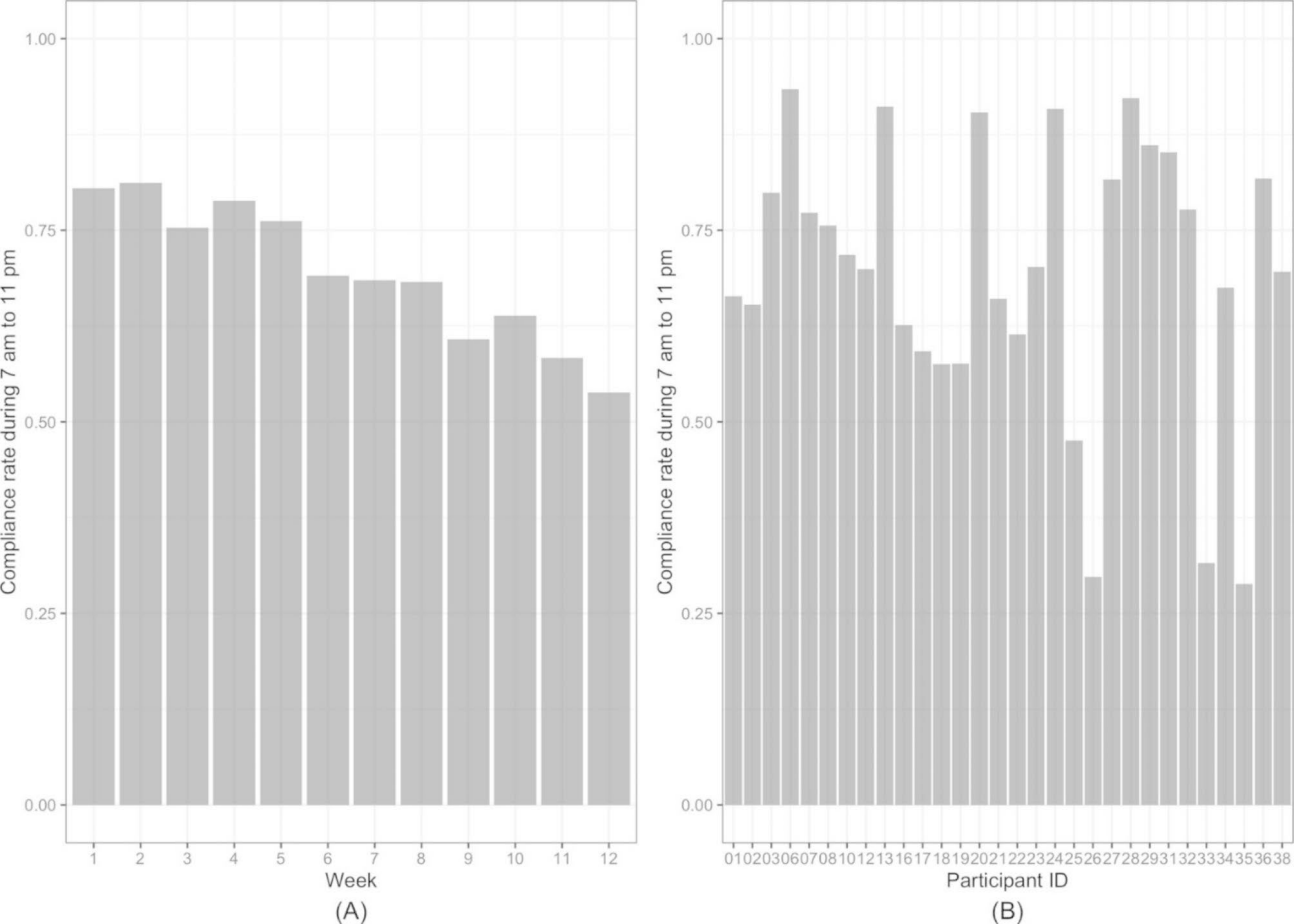
Compliance rate of wearing the Fitbit device from 7 am to 11 pm. (**A**) Average Fitbit compliance rates of all participants in different weeks. (**B**) Average Fitbit compliance rates of each participant in the study

In total, 30 participants (75%) were included in the Fitbit analysis. Of the remaining 10 participants, eight dropped out of the study, one participant’s Fitbit data were missing due to the replacement of the device, and one participant reported unexpectedly high physical activity (outlier) and was excluded from the analysis. Figure 2 displays the average Fitbit compliance rate from 7 am to 11 pm for each week of the study and for each participant. A decreasing trend in the compliance rate was observed over time (Fig. 2A), from 80.5% at week 1 to 53.8% at week 12. The overall compliance rate from 7 am to 11 pm varied among the participants (Fig. 2B), ranging from 28.9 to 93.4%. The average compliance rate of all participants was 69.5%. Nearly 83% of the participants reported that the feedback provided by Fitbit was helpful, while 58.6% reported that Fitbit helped them achieve their pain self-management goals. Approximately 65% of participants found it easy to use the Fitbit device in pain self-management, and 71% recommended using it to increase physical activity. The overall level of satisfaction with the Fitbit device in pain self-management was rated as satisfied by 89.6% of the sample.

### Biospecimen collection

A total of 33 blood samples (82.5%) were collected at the baseline visit and 25 blood samples (62.5%) were collected at the final visit. We collected buccal cell samples from seven participants because of small or hard-to-find veins. Therefore, 80% of the participants (n = 32) completed the biomarker assessment.

### Intervention efficacy

**Fig. 3:**
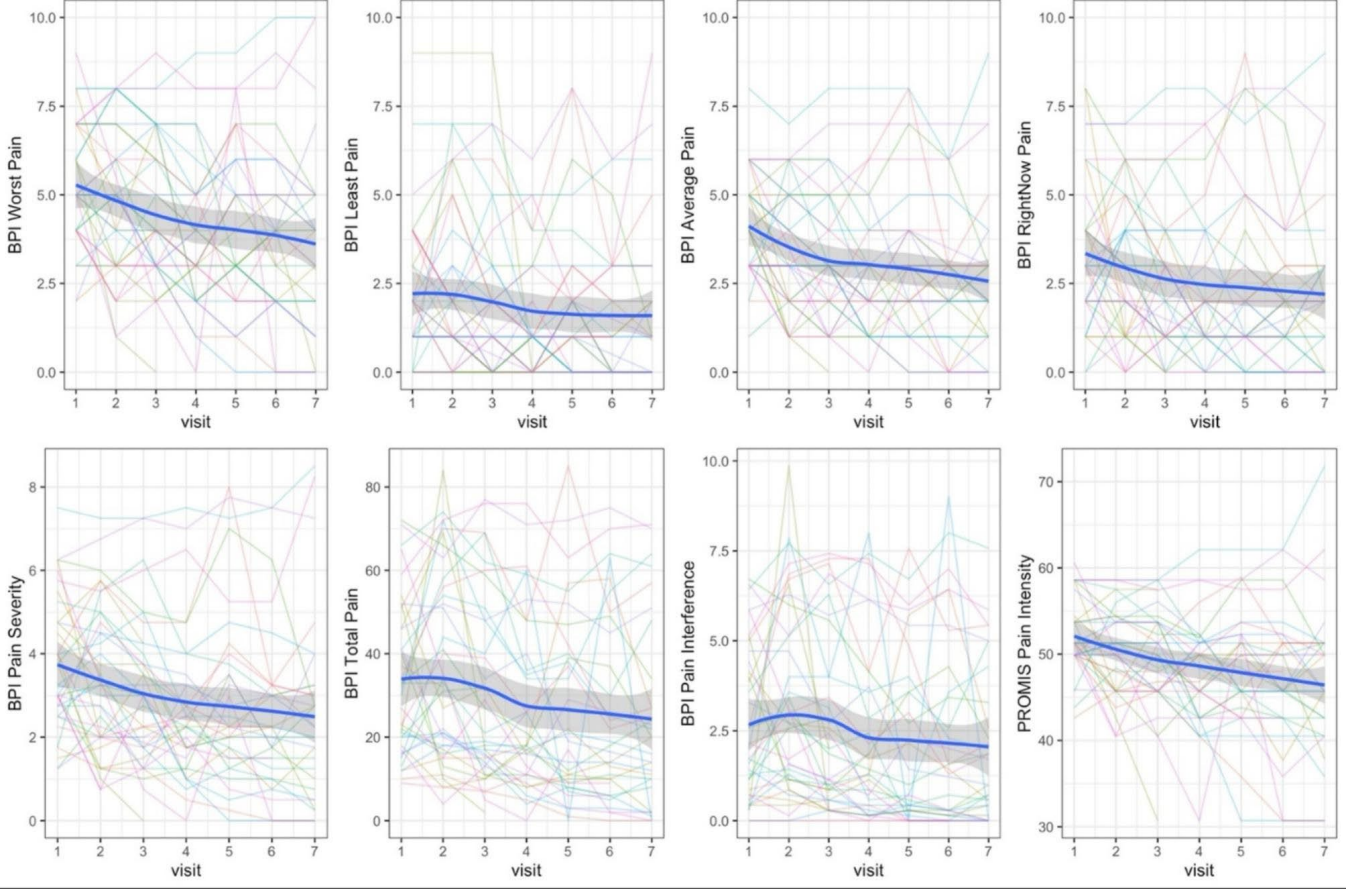
Subject trajectories and mean curves of pain measurement Abbreviations: BPI, brief pain inventory; PROMIS, patient-reported outcomes measurement information systems

We observed decreasing trends in the intensity of cLBP after participants received the PROPEL intervention. Figure 3 presents the subject trajectories and the decreasing sample mean curves of BPI worst pain, BPI least pain, BPI average pain, BPI right now pain, BPI pain severity, BPI total pain, BPI pain interference, and PROMIS-Pain Intensity.

Table 2 shows the results of the two-sample paired t-test used to detect the mean difference in pain outcomes between the baseline and 12-week follow-up visits. Participants reported significantly decreased BPI worst pain (d = -1.68, p = 0.003), BPI average pain (d = -1.61, p < 0.001), BPI right now pain (d = -0.97, p = 0.020), BPI pain severity (d = -1.20, p = 0.004), BPI total pain (d = -10.23, p = 0.004), BPI pain interference (d = -0.77, p = 0.020), and PROMIS-Pain Intensity (d = -5.61, p = 0.002) between pre- and post-intervention. Cohen’s D values indicated medium to large effect sizes for BPI worst pain (D = 0.57), BPI average pain (D = 0.77), BPI pain severity (D = 0.56), BPI total pain (D = 0.54), and PROMIS-Pain Intensity (D = 0.62).

**Table 2:** Results of two sample paired t-test for pain outcomes (N = 31)

Pain outcomes	Visit 1 Mean (SD)	Visit 7 Mean (SD)	Mean Difference (d)	p-value	Cohen’s D
BPI Worst Pain	5.25 (1.80)	3.61 (2.64)	-1.68	0.003	0.57
BPI Least Pain	2.19 (2.10)	1.65 (2.30)	-0.55	0.239	0.21
BPI Average Pain	4.19 (1.55)	2.61 (2.11)	-1.61	< 0.001	0.77
BPI RightNow Pain	3.19 (2.22)	2.23 (2.25)	-0.97	0.020	0.44
BPI Pain Severity	3.70 (1.63)	2.52 (2.23)	-1.20	0.004	0.56
BPI Total Pain	34.06 (18.77)	24.07 (22.19)	-10.23	0.004	0.54
BPI Pain Interference	2.75 (2.15)	2.00 (2.16)	-0.77	0.020	0.43
PROMIS Pain Intensity	52.12 (4.59)	46.54 (8.87)	-5.61	0.002	0.62

Abbreviations: BPI, brief pain inventory; PROMIS, patient-reported outcomes measurement information systems; SD, standard deviation

Table 3 shows the changes in the 12 QST measurements from the baseline visit to the last visit on both the control site and the pain site (n = 32). Only the warm detection threshold (WDT) at the pain site significantly increased (d = 0.26, p = 0.029) between the two visits, which showed that the participants’ sensitivity to detecting warm temperatures at the pain site increased after the PROPEL intervention.

**Table 3:** Results of two sample paired Wilcoxon sign-rank test for QST outcomes (N = 32)

QST		Visit 1 Mean (SD)	Visit 7 Mean (SD)	Mean difference	p-value
Mechanical detection threshold (mN)					
	Control site	3.04 (0.24)	3.10 (0.35)	0.06	1.000
	Pain site	3.21 (0.52)	3.24 (0.43)	0.03	0.466
Mechanical pain threshold (mN)					
	Control site	6.14 (0.48)	6.25 (0.44)	0.10	0.132
	Pain site	6.00 (0.45)	6.13 (0.43)	0.13	0.071
Mechanical pain sensitivity (pain rating 0–10)					
	Control site	1.96 (1.79)	1.91 (1.67)	-0.05	0.617
	Pain site	2.66 (2.11)	2.44 (1.72)	-0.22	0.439
Dynamic mechanical allodynia (pain rating 0–10)					
	Control site	0.76 (1.19)	0.76 (1.03)	0.11	0.452
	Pain site	0.84 (1.38)	0.73 (0.97)	-0.05	0.975
Windup ratio (multiple average/single average)					
	Control site	2.35 (5.21)	1.57 (1.37)	-0.78	0.899
	Pain site	2.57 (3.98)	2.32 (3.96)	-0.25	0.766
Vibration detection threshold (sec)					
	Control site	9.64 (3.13)	10.12 (3.08)	0.32	0.516
	Pain site	6.71 (4.66)	7.12 (3.89)	0.43	0.765
Heat Limits (°C)					
	Control site	43.31 (3.67)	42.51 (3.38)	-0.80	0.651
	Pain site	41.38 (3.48)	41.30 (3.10)	-0.08	0.919
Cold detection threshold (°C)					
	Control site	28.58 (2.22)	28.14 (2.55)	-0.44	0.304
	Pain site	28.60 (1.43)	28.40 (1.12)	-0.20	0.304
Warm detection threshold (°C)					
	Control site	35.38 (1.37)	35.70 (1.64)	0.32	0.477
	Pain site	35.82 (1.79)	36.08 (1.35)	0.26	0.029
Cold pain threshold (°C)					
	Control site	18.01 (9.90)	19.32 (8.12)	1.30	0.599
	Pain site	18.63 (10.05)	20.24 (8.08)	1.61	0.583
Heat pain threshold (°C)					
	Control site	40.94 (3.83)	41.51 (3.09)	0.57	0.410
	Pain site	40.65 (3.20)	40.69 (3.21)	0.03	0.978
Pressure pain threshold (kPa)					
	Control site	242.79 (120.25)	228.51 (140.43)	-14.28	0.239
	Pain site	252.6 (128.6)	277.22 (175.06)	24.62	0.360

**Abbreviations**: QST, quantitative sensory testing; SD, standard deviation

**Fig. 4:**
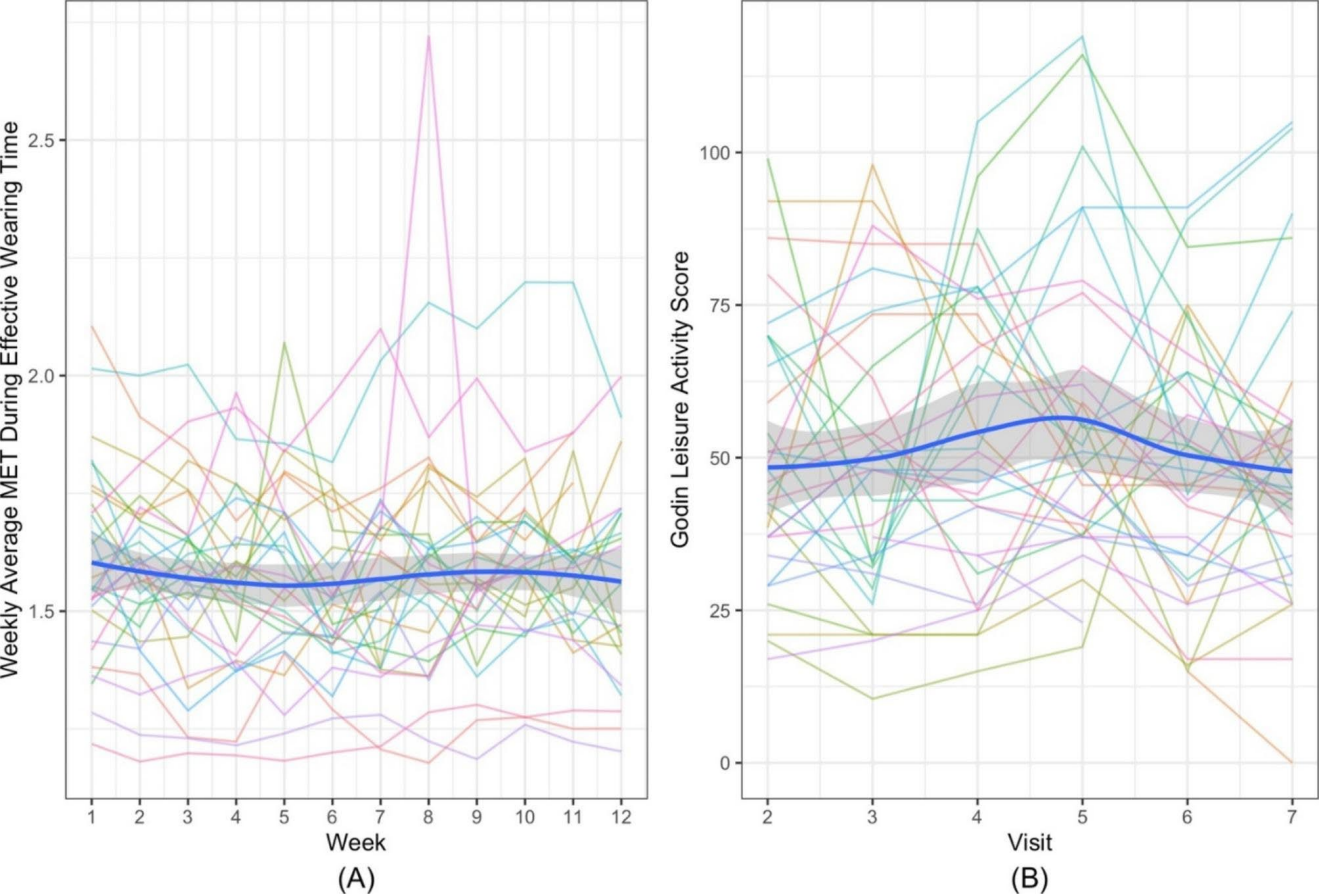
Subject trajectories and mean curves of Fitbit and Godin-Leisure physical activity measurements. (**A**) Weekly average MET during the effective wearing time of each participant. (**B**) Longitudinal Godin-Leisure activity scores of each participant. Each trajectory represents one participant’s longitudinal measurements during the PROPEL study, and the blue curves represent the average level of all participants Abbreviations: MET, metabolic equivalent; PROPEL, Problem-Solving Pain to Enhance Living Well

Figure 4 A and 4B display the trajectory plots of the weekly average MET and Godin-Leisure activity scores, respectively. There was no clear increasing trend in physical activity levels over the 12 weeks of the study.

## Discussion

We demonstrated the acceptability and feasibility of an Internet-based dissemination of pain self-management video modules and multidimensional data collection from adults with cLBP. Using the REDCap links, participants completed 10 short video modules and provided self-reported data, including self-management variables and patient satisfaction. Most participants wore an activity tracker with no pain or discomfort, and an online monitoring and storage system (Fitabase) using de-identified data was used to objectively measure physical activity levels. Participants’ satisfaction with PROPEL, including activity tracking and nurse consultation, was reasonably high. We observed acceptable retention and completion/response rates for the intervention protocol.

The response rate of self-reported surveys and wearable activity tracking technology in our sample was comparable to that of other studies among people with cLBP [20–22]. Overall, the success of the protocol may have resulted from the level of participant training and the detailed information provided at baseline visits. It should be noted that approximately 40% of the sample were university students. Future research should investigate strategies to effectively reach out to diverse subgroups of people with cLBP who may face challenges in participating in clinical trials. Most participants reported that wearing the activity tracker for over three months in the Fitbit satisfaction survey was not challenging. Our study’s definition of valid activity tracking data was comparable to that of studies commonly defining approximately 10–12 h of valid activity data as acceptable [23, 24].

Both baseline and 12-week follow-up visits involved surveys, QST measurements, and venipuncture for genetic markers. Despite the perceived concerns of participant burden, they were generally favorable toward our study protocol, including QST measures that involve noninvasive techniques to characterize pain phenotypes and can offer tailored exercise strategies [6]. Further research warrants describing pain phenotypic profiles and if and how exercise-based self-management interventions can change ones’ pain phenotypes in a large-scale randomized controlled trial. Of the 32 participants who visited the research suite for a 12-week follow-up period, only 25 blood samples were collected, which may be associated with the participants’ physiology and the research team’s experience. Our success rate of peripheral intravenous catheter insertion was 78.1% (25/32), slightly higher than studies reporting rates from 65 to 73% in the emergency department [25] and up to 65% in the hospital medical ward [26, 27].

We successfully delivered 10 short video modules focusing on pain physiology and pain management strategies, such as deep breathing and relaxation. Participants reported that receiving links (video URLs) for modules was convenient and helped them complete the modules based on their schedule. Existing studies mainly used REDCap links to collect self-reported data [28]; our study successfully disseminated video modules and tracked participants’ activities, enhancing the fidelity of our study protocol.

### Challenges and lessons learned

We acknowledge some challenges experienced in conducting this study. First, although the intensive data collection schedule was communicated during the consent procedures, not all participants were able to engage throughout the data collection process. The research team made substantial efforts to set up and execute the study protocol, from scheduling baseline visits to collecting patient satisfaction data and following up with the participants. Researchers and clinicians in regions with limited resources might be cautious about the implementation of the PROPEL intervention due to the intensive multidimensional data collection. However, continued research on pain phenotyping can simplify QST measurements and generic markers needed for the patient classification for tailored interventions among individuals with cLBP.

Bi-weekly nurse consultations, in particular, need consideration to accommodate each participant’s course and work schedule to avoid deviating from the study protocol. The maximum number of contact attempts was set *a priori* as a limit of three times over 2–3 day intervals. In some cases, we failed to retain participants despite multiple attempts. Innovative strategies to efficiently maintain high retention rates, such as using social media or a study Internet site, have been discussed [29]. Social media has recently been considered as a platform for disseminating research information and keeping participants engaged. We must also acknowledge the importance of understanding the target population’s characteristics, emphasizing study benefits and commitments, including expectations, and being flexible in accommodating participants’ needs [30].

Data recorded by Fitbit device needed additional data processing procedures to achieve an appropriate analysis. Due to the limitation of Fitbit Flex 2, true sedentary time, sleeping time, and unoccupied time were all recorded as sedentary time in the Fitbit database. If a participant did not wear the Fitbit device continuously, the duration of different levels of active time recorded by Fitbit Flex 2 did not reflect the true activity variability. Since this type of “missing” data cannot be identified in the Fitbit data set, the statistical missing data algorithm cannot be applied. Therefore, we calculated the average MET level on the approximated effective wearing time to obtain a fair comparison of physical activity across the participants. More accurate and reliable measurements of physical activity are required for future studies based on data collected using Fitbit Flex 2.

The inter-and intra-rater reliability of the QST protocol is acceptable for determining somatosensory abnormalities in multiple areas [31–33]. Additionally, we conducted a series of hands-on trainings in QST, and written protocols were available to all research team members. These efforts made QST measurements feasible in this study, and only one participant declined the QST at the 12-week follow-up.

The participants’ satisfaction with the video modules offered by the REDCap links was high. Using the REDCap system, we tracked when each participant started watching the video module. A nurse research staff member also invited the participants to discuss the video modules and self-management barriers during the consultation. However, as in other studies using online modules, we could not monitor participants’ retention of information. Technology-based interactive modules, such as online quizzes, drag-and-drop activities, and game-type activities, can be considered to enhance participants’ learning experiences.

### Implications and contributions to research and practice

Pain is a complex condition involving bio-psychosocial factors that require multidimensional assessment and personalized management to improve health outcomes. Due to the refractory nature of non-specific cLBP, a self-management program that often involves intensive education and training is crucial for empowering patients to manage their pain. Multidimensional assessment of the PROPEL intervention using biospecimen collection, wearable activity tracking technology, and the REDCap system appeared to be feasible. Self-management interventions delivered via technology have great potential to reach diverse, possibly hard-to-reach populations and offer personalized pain self-management interventions by integrating pain phenotypes, genetic markers, and physical activity types affecting pain conditions.

## Conclusions

This one-arm longitudinal study demonstrated adequate feasibility and acceptability of the PROPEL intervention and research protocol, and preliminary efficacy for improving cLBP outcomes. Additional research is needed to integrate strategies for increasing physical activity and measurement over time in people with cLBP, as well as a clinical trial of the PROPEL intervention with a control group to determine its effectiveness in a larger sample. As more robust evidence is needed to identify the most effective components of pain self-management for cLBP, this study is the first step in contributing to the evidence base. Overall, the results are promising and support continued research on PROPEL self-management interventions for individuals with cLBP.

## Data Availability

The data will be available upon reasonable request. Please contact the corresponding author for data requests.
